# Peritonitis – the Eastern experience

**DOI:** 10.1186/1749-7922-1-13

**Published:** 2006-04-26

**Authors:** Sanjay Gupta, Robin Kaushik

**Affiliations:** 1 Department of Surgery Government Medical College and Hospital Chandigarh, India

## Abstract

Peritonitis is a common emergency encountered by surgeons the world over. This paper aims to provide an overview of the spectrum of peritonitis seen in the East. Studies dealing with the overall spectrum of secondary peritonitis in various countries of this region were identified using Pubmed and Google. These were analyzed for the site and cause of perforation and the mortality. It was observed that perforation of duodenal ulcers was the most the commonly encountered perforations. These are followed by small bowel and appendicular perforations. Colonic perforations were uncommon. The overall mortality ranges between 6–27%.

## Background

Despite a better understanding of pathophysiology, advances in diagnosis, surgery, antimicrobial therapy and intensive care support, peritonitis remains a potentially fatal affliction. On the basis of source and nature of the microbial contamination peritonitis can be classified as primary, secondary and tertiary. Primary peritonitis is infection, often monomicrobial, of the peritoneal fluid without visceral perforation. Secondary peritonitis arises subsequent to loss of integrity of a hollow viscus and is the most common form of peritonitis encountered. Tertiary peritonitis develops following treatment of secondary peritonitis either due to failure of the host inflammatory response or due to superinfection [1]. The contamination of peritoneal cavity thus, can lead to a cascade of infection, sepsis and multisystem organ failure (MSOF) and death if not treated in a timely manner.

This paper aims to present the experience of the East in terms of the spectrum of secondary peritonitis seen, its common causes and outcomes.

## Materials and methods

A search of available English Language Literature was conducted to identify studies dealing with secondary peritonitis, predominantly from Asia, using Pubmed and Google. The key words 'peritonitis' and 'perforation' were used as a basic level of search. The results thus obtained were further refined using the names of the various countries of this region in an effort to identify such series that dealt with the overall spectrum of peritonitis as seen in the different geographical locations within this region. These studies were further cross referenced to screen for any other relevant series in their bibliography. Using these methods, a total of fifteen series were identified that dealt with the overall spectrum of peritonitis. These were then analysed for the total number of cases, the distribution of the site of perforation and the overall mortality. A few series were not considered truly representative of the overall spectrum of peritonitis and were therefore, not included for analysis.

A second search was similarly carried out to identify studies that dealt with perforations of specific anatomical parts of the bowel, the underlying pathology, and the preferred methods of their treatment and mortality.

The results are tabulated as Table 1 (overall spectrum), Table 2 (Gastroduodenal perforations) and Table 3 (Small bowel perforations).

**Table 1:** Previously reported series of peritonitis

**Author [Ref]**	**Total Cases**	**Gastroduodenal Perforations n (%)**	**Small bowel Perforations n (%)**	**Appendicular Perforations n (%)**	**Colorectal Perforations n (%)**	**Mortality**
Quereshi 2005[16]	126	31 (24.6)	37 (29.4)	12 (9.5)	3 (2.4)	15%
Khan 2004[11]	54	21 (38.8)	14 (25.9)	6 (11.1)	4 (7.4)	NA
Nishida 2002*[15]	229	92 (40.2)	71 (31)	0	66 (28.8)	13.1%
Chen 2000[14]	98	57 (58.1)	6 (6.1)	13 (13.2)	14 (14.3)	NA
Dorairajan 1995[2]	250	80 (32)	103 (41.2)	38 (15.2)	5 (2)	9.2%
Shreshtha 1993[12]	80	26 (32.5)	15 (18.7)	27 (33.7)	0	9.6%
Tripathi 1993[3]	160	24 (15)	57 (35.6)	16 (10)	NA	23.7%
Dandpat 1991[4]	340	276 (81.1)	34 (10)	22 (6.4)	4 (1.2)	15.9%
Sharma 1991[5]	155	47 (30.3)	62 (40)	23 (14.8)	2 (1.3)	8.4%
Shah 1988[6]	110	51 (46.4)	16 (14.5)	31 (28.1)	3 (2.7)	6.4%
Kachroo 1984[7]	90	15 (16.6)	13 (14.4)	37 (41.1)	2 (2.2)	8.8%
Rao 1984[8]	46	26 (56.5)	18 (39.1)	2 (4.3)	0	26.1%
Ratnatunga 1983[13]	131	11(8.3)	31(23.7)	15(11.4)	NA	NA
Bhansali 1967[10]	96	48 (50)	40 (41.6)	-**	0	20.8%

n – number of cases

NA – data not available

* – includes traumatic perforations

** – not included in the study

**Table 2:** Summary of previously reported series of gastroduodenal perforations

**Author [Ref]**	**Cases**	**Duodenal Ulcer Perforation n (%)**	**Gastric ulcer Perforation n (%)**	**Perforation of Gastric Carcinoma n (%)**	**Mortality**
Khan 2004[11]	21	16 (76.2)	5 (23.8)	0	NA
Siu 2001[25]	121	83 (68.6)	29 (23.9)	-*	3.3%
Chan 2000[17]	206	196 (95.1)	10 (4.8)	0	10.7%
Dorairajan 1995[2]	80	74 (92.5)	5 (6.2)	1 (1.2)	3.7%
Sugimoto 1994[60]	101	90 (89.1)	11 (10.8)	0	0
Wakayama 1994[18]	136	110 (80.9)	19 (13.9)	7 (5.1)	5.1%
Sharma 1991[5]	47	45 (95.7)	1 (2.1)	1 (2.1)	4.2%

n – number of cases

NA – data not available

* – not included in the study

**Table 3:** Summary of previously reported series of small intestinal perforations

**Author [Ref]**	**Total Cases**	**Typhoid Perforations n (%)**	**Nonspecific Ulcer Perforation n (%)**	**Tubercular Perforations n (%)**	**Mortality**
Khan 2004[11]	18	7 (38.9)	5 (27.8)	2 (11.1)	NA
Chaterjee 2001, 2003[28,34]	460	248 (53.9)	111 (24.1)	16 (3.5)	20.9%
Chitkara 2002[65]	216	92(42.6)	36(16.7)	36(16.7)	11.5%
Ray 2001[61]	30	8(26.7)	5(16.7)	4(13.3)	6.7%
Chulakamontri 1996[20]	8	2 (25)	1 (12.5)	0	0
Dorairajan 1995[2]	103	69 (66.9)	7 (6.8)	13 (12.6)	NA
Sharma 1991[5]	62	42 (67.7)	5 (8.1)	12 (19.3)	11.3%
Bose 1986[62]	75	46 (61.33)	1 (1.3)	8 (10.6)	16%
Khanna 1984[63]	125	100 (80)	0	4 (3.2)	NA
Nadkarni 1981[35]	32	8(25)	18(56.2)	3(9.3)	28.1
Mehendale 1979[64]	32	9(28.1)	2(6.2)	13(40.6)	37.5%
Bhansali 1967[10]	46	29 (63)	0	7 (15.2)	NA

n – number of cases

NA – data not available

## Results

Despite peritonitis being a commonly encountered surgical emergency, we could identify only fifteen series in the available English Language Literature that dealt with the overall spectrum of secondary peritonitis from Asia and far East. The majority of these were from India [2-10], with only two from Nepal [11,12] and one each from Srilanka [13], China [14], Japan [15] and Pakistan [16]. The full text of two could not be obtained for analysis [9,13].

Overall, perforations of the gastroduodenum are the most common cause of peritonitis. [Table 1]. These commonly arise consequent to perforation of peptic ulcers, more specifically; ulcers of the first part of the duodenum [Table 2]. The mortality in this subset of perforations is upto 11 %, with a higher mortality seen in patients over the age of 50 years and in those who present late to the hospital [17-19].

The next commonly encountered perforations arise from the small bowel (6–42 %) [Table 1], but there seems to be a wide geographical variation in the incidence and frequency of small bowel perforations. A few authors have reported this to be the most common cause of peritonitis in their series [2,3,5,13,16], whereas it accounted for only around 6 % of the cases of peritonitis that were encountered in China [14]. A very low incidence of small bowel perforations has also been reported from Thailand [20]. Generalized peritonitis due to perforation of the small bowel is seen more commonly in the developing countries, where it is usually secondary to perforation of typhoid ulcers that are seen in enteric fever. Non specific or idiopathic ulcer perforation and tubercular ulcer perforations are the next common cause in most of the series [Table 3]. The overall mortality in this group of peritonitis is higher than that seen in gastroduodenal ulcer perforations, and ranges from 0 – 38 % [Table 3].

These two types of perforations accounted for the vast majority of cases of peritonitis encountered, although appendicular perforations are also frequently implicated [6,7,12,14]. Colorectal perforations are rare [Table 1] but a higher incidence has been reported from Japan and China [14,15]. The other rare causes of peritonitis that have been reported are perforations of the gallbladder, common bile duct, uterus, liver or splenic abscesses [2,5,7,11,13,16].

## Discussion

Although there is a paucity of data on the overall spectrum of peritonitis in the East, the few studies [Table 1] that we were able to identify commonly implicated perforations of the gastroduodenum as the commonest cause of peritonitis in this region; there definitely is a regional bias in the frequency and incidence of intestinal perforations, with enteric perforations being encountered more frequently in the developing countries of south East Asia, and colonic perforations in the far east.

### Gastroduodenal perforations

Perforations of peptic ulcers form the major group among the gastroduodenal perforations [Table 2]. These perforations are usually encountered along the first part of the duodenum anteriorly and in the pylorus of the stomach. The advances in the medical treatment of the peptic ulcer disease have led to a dramatic decrease in the number of elective surgeries performed. However, the number of patients undergoing surgical intervention for complications such as perforation remains relatively unchanged or has increased [21,22]. Such patients present with the classical signs and symptoms of peritonitis, and need early surgery for a favourable outcome. Although the surgical options are many – from simple closure to definitive acid reducing procedures – it has been our experience that simple closure of the perforation using a pedicled omental patch gives good results, even in large perforations upto 3 cms diameter [23]. This should therefore, be the preferred surgical method of closure, as it is easy to perform, is technically straightforward, and gives comparable results to that of definitive surgery [23,24]. The mortality rate of these perforations varies from 4 – 11% [17-19,23,25], and is higher in the elderly, those with concomitant disease, preoperative shock, larger size of the perforation, delay in presentation and delay in operation [17-19]. Perforation of ulcers at other sites within the stomach and gastric cancers has been uncommonly reported, and emergency gastrectomy is the treatment of choice [26,27].

### Small bowel perforations

The next common types of perforations encountered are those arising in the small intestine [Table 3]. These usually arise on a background of enteric fever, when the ulcerated peyer's patches in the terminal ileum perforate to give frank peritonitis. These typhoid ileal perforations have a high mortality rate, upto 60% [28-32]. Aggressive resuscitation, antibiotics and early surgery has reduced the mortality rate and complications in this subset of small bowel perforations to less than 10% [28]. Although early surgery is associated with a better outcome, there is, however, no uniformity of opinion about the operative procedure to be performed in these perforations, and various procedures have been described such as simple closure, wedge excision or segmental resection and anastomosis, ileostomy, and even, side to side ileo-transverse anastomosis after primary repair of the perforation [28-33]. However, it is our experience and teaching, that these patients have bowel oedema that precludes any suturing, and therefore, exteriorization of the perforation as a 'loop' ileostomy is the safest and fastest procedure to be done. Closure of this loop ileostomy is performed electively after 6 to 8 weeks, and is safe. A primary anastomosis (simple closure) is to be considered only when the patient presents early and the bowel is healthy.

A 'non specific' etiology is attributed to small bowel perforations when the perforation cannot be classified on the basis of clinical symptoms, gross examination, serology, culture and histopathological examination into any disease state such as enteric fever, tuberculosis or malignancy [20,34,35]. These ulcers are usually single and commonly involve terminal ileum [34]. It has been proposed that submucus vascular embolism [36], chronic ischemia due to atheromatous vascular disease or arteritis [37], or drugs such as enteric coated potassium tablets [38] are responsible for them.

These 'non specific' ileal perforations are closely followed by small bowel perforations occurring in intestinal tuberculosis. Most of these (50 – 80 %) occur in the ileum, usually proximal to strictures of the bowel [39]. Free tubercular perforations are rare [40]. The mortality rate reported in these tubercular perforations is very high, upto 70 % [41]. The diagnosis of perforated tubercular enteritis is usually not one that is made pre-operatively, because of the non-specific signs and symptoms and absence of radiological evidence of tuberculosis in the chest. Even in the presence of tubercular lesions in the chest skiagram, the diagnosis is not entertained or established until the histology and culture of the biopsied tissue turns out to be positive [42]. The recommended treatment after source control is multidrug anti tubercular therapy [43].

In contrast to these common causes of small bowel perforation in the developing countries, small bowel perforations are rare in the oriental countries [14,20]. Apart from enteric fever and 'non specific' ulcers [20,44], the other reported causes of such perforations from these countries include Crohn's disease, Behcet's disease, radiation enteritis, adhesions, ischemic enteritis, SLE and very rarely, intestinal tuberculosis [20,45-47]. Free perforations are a rare complication of Crohn's disease, and their incidence is reportedly highest from Japan, where it ranges from approximately 3% to 10%. These perforations are usually solitary, and occur mainly in the ileum. However, they can be multiple, and can occur at any site in the small or large bowel [45]. Similarly the incidence of Behcet's disease is much higher in Japan, and perforation of the intestinal ulcers can occur in upto 56% of cases. These are usually multiple and occur commonly in the terminal ileum and caecum, and need removal of a long segment of the ileum to prevent post-operative recurrence [46].

### Appendicular and colorectal perforations

Gastroduodenal and small bowel perforations form the majority of cases encountered. Few series have shown higher or equal incidence of appendicular perforations [7,21]. However, this high incidence of appendicular perforations probably reflects the younger age of patients in the reported series where appendicitis and consequently the complications are known to be much higher [7]. Colorectal perforations are uncommon, and apart from occasional case reports, we could come across only a single series from Japan that dealt with non appendicular colorectal perforations [48]. Perforations secondary to colonic neoplasms account for the majority of such cases. The perforation may occur at the site of the malignancy or proximally, as a 'blow out' of the proximal large bowel due to obstruction from the lesion. The incidence of such perforations is low, but carries a high mortality of about 17% [48,49].

The other causes that have been reported are perforation of colonic diverticula, inflammatory conditions of the colon, volvulus, mesenteric ischemia, trauma, iatrogenic complications, idiopathic and stercoral perforations [48,50]. In the Asian communities diverticular disease is more common in a younger age group, and the right colon is more commonly involved. One-third of these patients present with perforation of the large bowel and fecal peritonitis that requires surgical intervention [51,52]. Amoebic colitis is another condition that is common in the tropical countries, with an incidence of perforation around 2 %, but with a high mortality rate (up to 50%) regardless of the treatment [53].

### Rare perforations

Rare sites of perforation leading to secondary peritonitis that have also been reported in the literature arise from the biliary tree, uterus, splenic and liver abscesses. Of these, ruptured amoebic liver abscesses are frequently encountered in tropical countries. These are seen in 3–7% of cases of intestinal amoebiasis, and upto 22% can rupture to give peritonitis, which carries a high mortality [54]. The management is by laparotomy and drainage or non-operatively, by means of metronidazole and/or radiologically guided drainage [54,55]. Rupture of pyogenic liver abscess is rarer by comparison [56].

### Spectrum of bacterial isolates

The bacterial analysis of the peritoneal fluid encountered showed E. coli to be dominant pathogen isolated, ranging from 25–71% [3,6,7,10,57,58]. This was followed by Bacteroides fragilis, [57,58] Klebsiella sp., and Pseudomonas sp [6,7], in the few series that dealt with this aspect of peritonitis. A sterile culture was encountered in 8–59.1% [3,6,10,58]. Depending on the site of the perforation gram positive cocci are predominantly isolated in gastroduodenal perforations; Pseudomonas sp. in small bowel perforations; and E.coli in appendicular and colonic perforations [12]. However, a higher incidence of fungal isolates has also been reported after gastroduodenal perforations [59].

## Competing interests

The author(s) declare that they have no competing interests.

## Authors' contributions

**SG **carried out acquisition, analysis, interpretation of the data and drafting of the manuscript.

**RK **was involved interpretation of the data, drafting of the manuscript, and revised it critically for the intellectual content till the final version was reached.

Authors have read and approved the final manuscript.
